# Thymic seminoma

**DOI:** 10.4103/0971-5851.56338

**Published:** 2009

**Authors:** J. Kannan, P. Karkuzhali, S. Lakshminarasimhan

**Affiliations:** *Department of Medical Oncology, Institute of Pathology, Madras Medical College, Chennai, India*

**Keywords:** *Thymic seminoma*, *anterior mediastinal mass*, *thoracotomy*

## Abstract

Thymic seminoma is a rare clinical entity. We report a case of an 18-year-old male patient who presented with chest pain, dyspnea, dysphagia of 1-month duration. Contrast-enhanced computer tomogram of chest showed an anterior mediastinal mass. He was subjected to thoracotomy and excision of the mass. Histopathology examination revealed seminoma of thymus. He failed to follow up for a period of 8 months post-surgery as he was asymptomatic. He then presented with a recurrence of the tumor locally, along with metastases to the lung, para-aortic and peri-aortic lymph nodes. He was kept on cisplatin, bleomycin and etoposide.

## INTRODUCTION

Seminoma of thymus should be regarded as a germ cell tumor rather than a true thymoma. It affects only young males. It grows very slowly and can become large before the appearance of symptoms. Differential diagnosis includes thymoma, lymphoma, ectopic thyroid, ectopic parathyroid, mesenchymal and neurogenic tumors. Chemotherapy plays a major role in the management of this neoplasm.

## CASE REPORT

**Figure 1:**
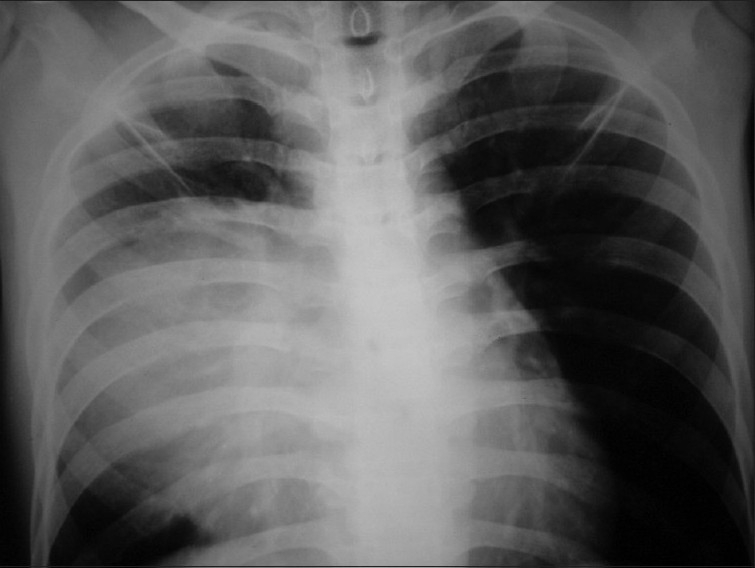
Chest X ray PA view (2006) shows homogenous opacity in right mid and lower zone

An 18-year-old male patient presented in September 2006 with dull retrosternal and right-sided chest pain, dyspnea and dysphagia for solids of 1-month duration. Chest X-ray showed lobulated homogenous opacity occupying the right mid-zone and lower zone [Figure 1]. There was no calcification.

**Figure 2:**
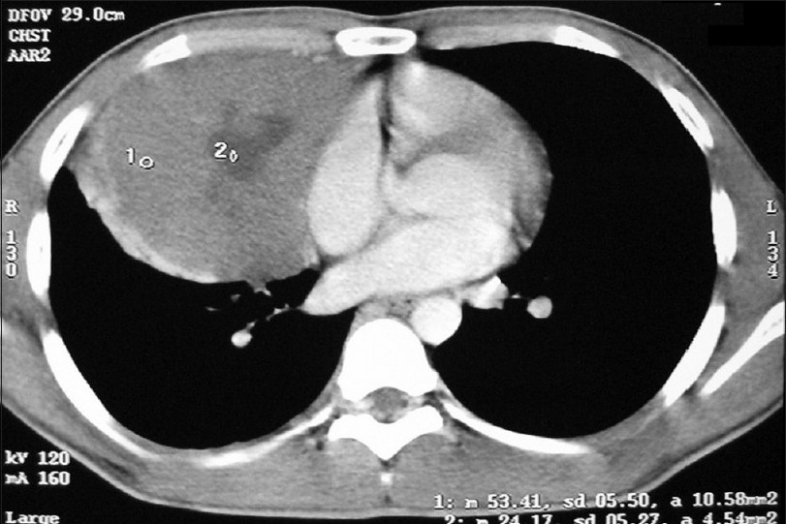
CT chest (2006 ) shows anterior mediastinal mass extending into right side of chest

**Figure 3:**
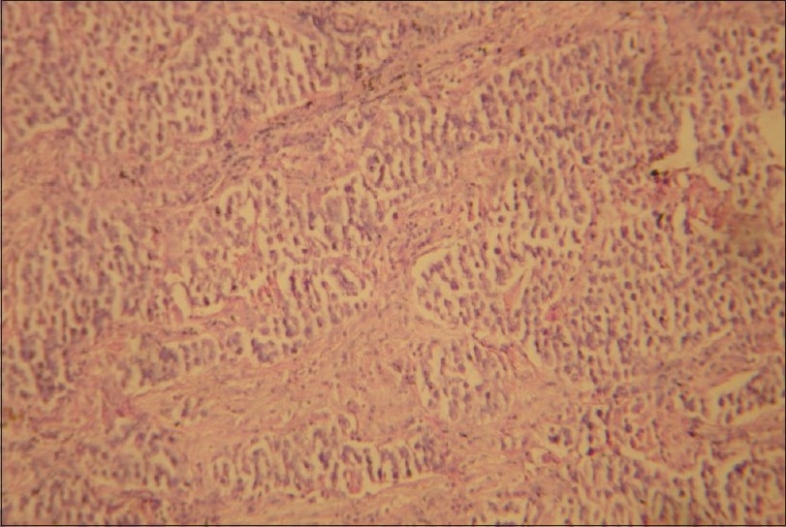
Low power view of thymic seminoma showing fibrous septae

**Figure 4:**
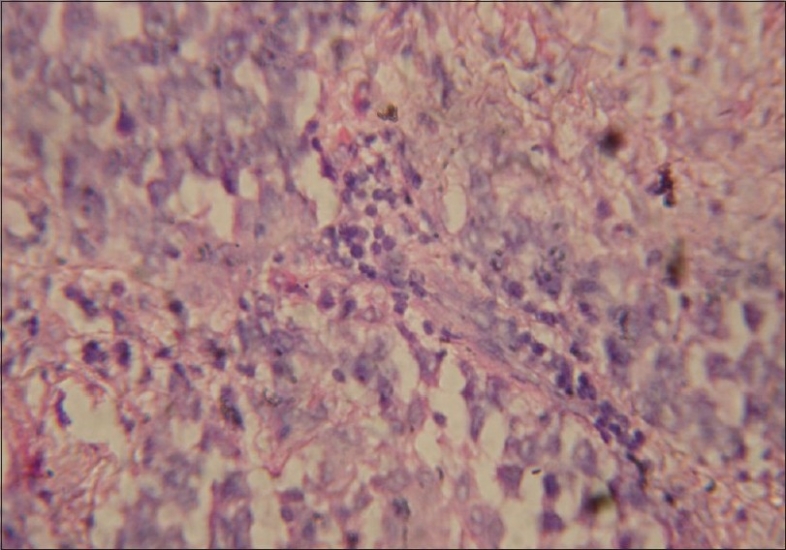
High power view of thymic seminoma showing fibrous septa infiltrated by lymphocytes and plasma cells, large amounts of cytoplasmic glycogen and an irregular skein like nucleolus

**Figure 5:**
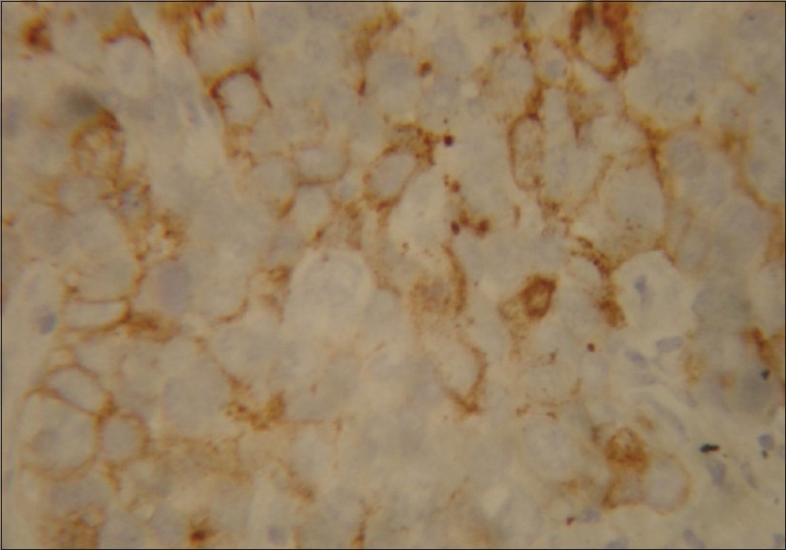
Immunohistochemistry showing placental alkaline phosphatase staining

CECT chest showed anterior mediastinal mass with extension into right side of chest [Figure 2]. Image-guided biopsy of the lesion was done. The results did not reveal malignancy. Cardiothoracic surgeons proceeded with right thoracotomy and excision of the mass. Histopathology of the specimen showed seminoma of thymus [Figures 3 and 4]. The result of the test for placental alkaline phosphatase was positive [Figure 5]. The patient failed to follow up for 8 months as he was asymptomatic.

**Figure 6:**
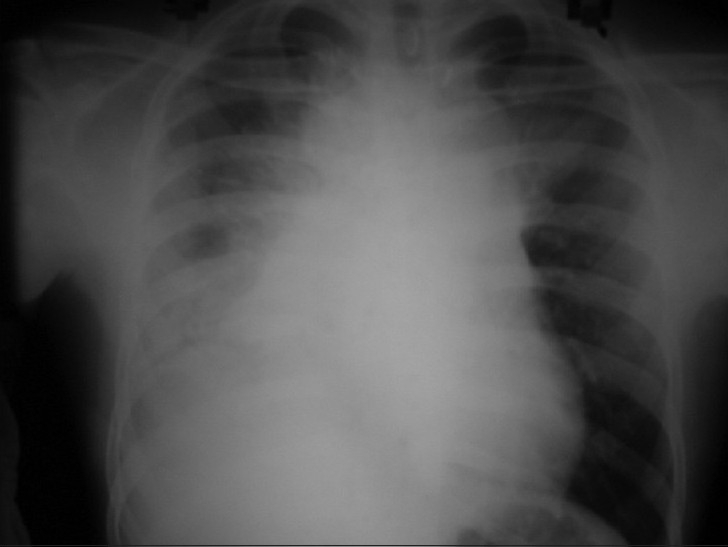
Chest X ray PA view (2007) shows superior mediastinal widening with homogenous opacity in right mid and lower zone

**Figure 7:**
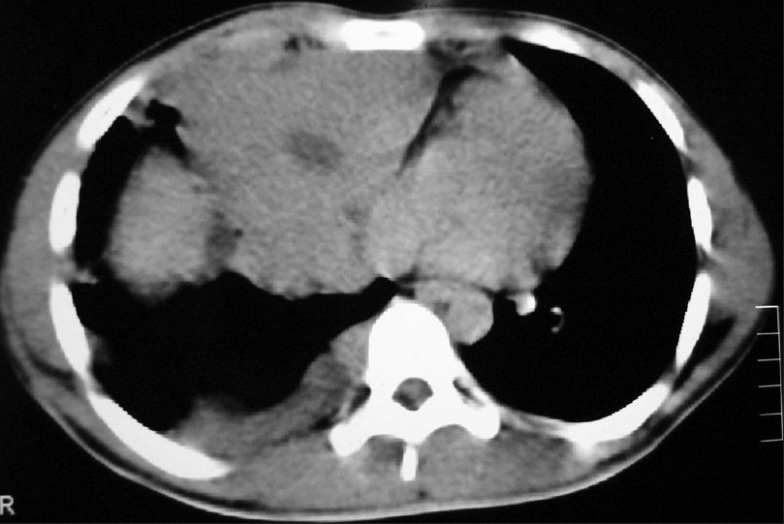
CT chest (2007 ) shows recurrence of anterior mediastinal mass extenting into right side of chest

He presented in June 2007 with dull-aching retrosternal and right-sided chest pain with exertional dyspnea and dysphagia for solids. On examination, there was impaired note to percussion over the right mammary and infra-axillary regions with diminished breath sounds. A 3 ×3 cm mass was palpable over the epigastric region. Both testes were normal. Chest X-ray showed superior mediastinal widening with homogenous opacity in the right mid and lower zone [Figure 6]. CECT chest showed an anterior mediastinal mass extending into middle mediastinum with subpleural metastases to posterior segment of right lower lobe [Figure 7].

**Figure 8:**
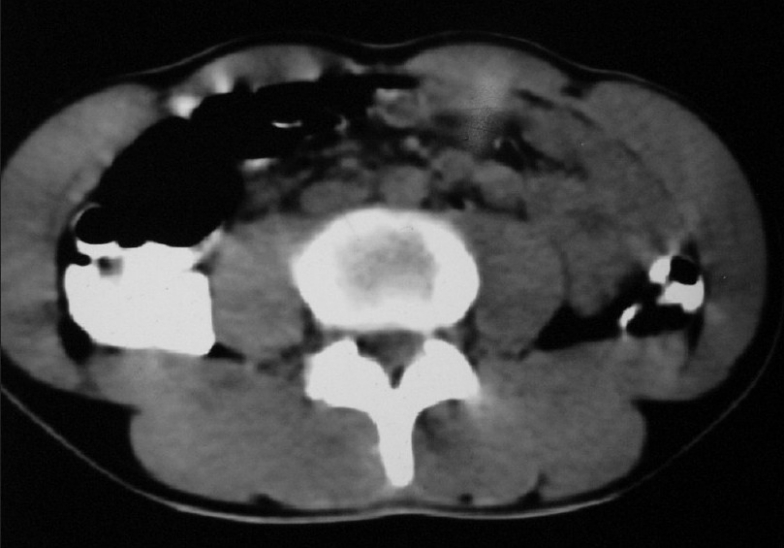
CT abdomen (2007) shows multiple para aortic lymphadenopathy

Contrast enhanced Computed tomography (CECT) abdomen showed extrinsic compression of liver by subpleural metastases with multiple para-aortic and peri-aortic lymphadenopathies [Figure 8]. USG (Ultrasonogram) of scrotum was normal. His serum tumor markers were alpha fetoprotein: 4.3 ng/mL beta human chorionic gonadotropin: 39.4 mIU/mL lactate dehydrogenase: 742 U/L.

**Figure 9:**
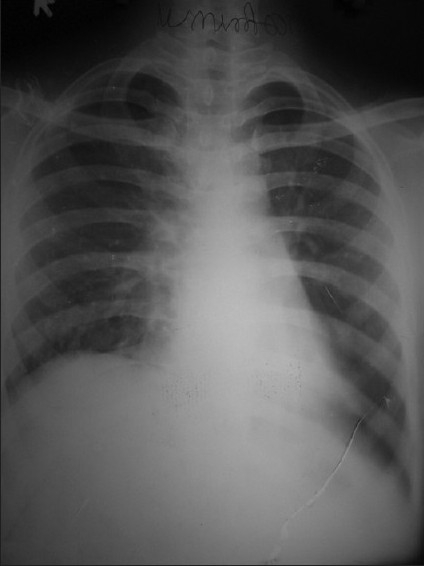
Chest X ray PA view (2008 - post chemo) - disappearance of mass

**Figure 10:**
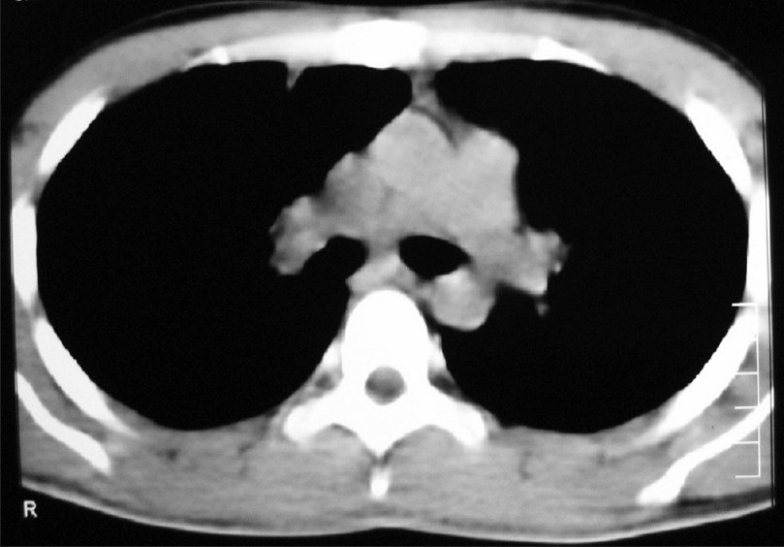
CT chest (2008) shows complete disappearance of the mass after chemo

**Figure 11:**
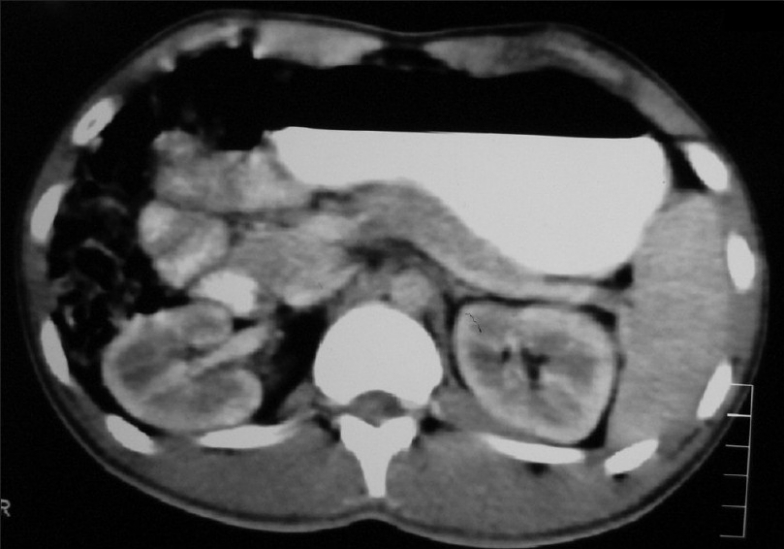
CT abdomen (2008) shows reduction of para aortic lymphnodes to sub centimeter size after chemo

The diagnosis of recurrent seminoma of thymus with metastases to right lung, para- and peri-aortic lymph nodes was made. The patient was started on BEP (bleomycin, etoposide, cisplatin). After four courses of chemo, complete disappearance of the mediastinal mass and lung metastatic lesions and reduction of abdominal lymph nodes to sub-centimeter size were noted [Figures 9–11].

## DISCUSSION

Malignant mediastinal germ cell tumors are uncommon, representing only 3% to 10% of tumors originating in the mediastinum.[1–3] They account for 2% to 5% of all germ cell tumors. They constitute 50% to 70% of all extragonadal germ cell tumors.[4] Other anecdotal sites of extragonadal germ cell tumors are retroperitoneum, pineal and suprasellar regions and very rarely vagina, prostate, liver, gastrointestinal tract and orbit.

Great majority of mediastinal germ cell tumors occur in patients between 20 and 35 years of age. Mature cystic teratoma is the most common benign mediastinal germ cell tumor and affects both sexes equally. Seminoma and non-seminomatous germ cell tumors show male predilection.

Mediastinal germ cell tumors arise as a consequence of abnormal migration of germ cells during embryogenesis.[5] The cytogenetic finding of one copy or multiple copies of the short arm of chromosome 12p with loss of the long arm of chromosome 12 is seen in nearly all germ cell tumors.[6,7]

The morphologic appearance is identical to that of its testicular counterpart at the light microscopic, immunohistochemical and ultrastructural levels.[8,9] Focal weak staining with *p53* IHC was observed in 31% of mediastinal seminomas and 77% of testicular seminomas. Eight percent of mediastinal seminomas showed *K-ras* mutation (codon 13 GGC > GAC; glycine > aspartate), which is in contrast to 15% of testicular seminomas showing codon 12 mutations. Therefore, primary mediastinal seminomas appear to be different in their *K-ras* sequence and *p53* immunostain profile from testicular seminomas. Codon mutation type may be useful in determining primary versus metastatic origin of a mediastinal seminoma.[10]

The presence of fibrous septa infiltrated by lymphocytes and plasma cells, epitheloid granulomas, germinal centers, large amounts of cytoplasmic glycogen and an irregular skein-like nucleolus favors the diagnosis of seminoma. The true nature of the lesion may be obscured by the presence of a very prominent granulomatous reaction, reactive follicular hyperplasia, epithelium-lined cystic formation of thymic origin and fibrosis.[11]

It is immunoreactive for placental alkaline phosphatase, CD 117 and often CD 57, but negative for CD 45 and usually negative or focally positive for cytokeratin.[12]

Approximately 20% to 35% of seminomas are detected by routine chest radiography while still asymptomatic. The most common initial symptom is a sensation of pressure or dull retrosternal chest pain. Other symptoms include exertional dyspnea, cough, dysphagia, hoarseness of voice. Superior vena cava (SVC) syndrome develops in approximately 10% of the patients.[13]

At the time of diagnosis, only 30% to 40% of patients with mediastinal seminoma have localized disease. The lungs and other intrathoracic structures are the most common metastatic sites. The skeletal system is the most frequently involved extrathoracic metastatic site, while the retroperitoneum is an uncommon site of metastases in these patients with mediastinal seminoma.

They appear as large anterior mediastinal mass without calcification in chest X-ray. A CT scan of the chest typically shows a large homogenous anterior mediastinal mass that obliterates the fat planes surrounding mediastinal vascular structures.

Elevated serum levels of β human chorionic gonadotropin are detected in approximately 10% of mediastinal seminomas. A level of serum β human chorionic gonadotropin exceeding 100 ng/mL is unusual. Serum alpha fetoprotein level is always normal in pure mediastinal seminoma. Serum lactate dehydrogenase is also elevated in the majority of patients with mediastinal seminoma.

If distant metastases or obviously unresectable intrathoracic tumor is present, a histological diagnosis should be made using the least invasive approach, because surgical therapy does not play a role in the initial treatment of these patients, and rapid initiation of definitive systemic therapy is essential. Patients with small tumors that appear resectable should undergo thoracotomy and attempted complete resection; postoperative radiotherapy 40 to 45 Gy is curative.

Pure mediastinal seminomas are curable in majority of the patients. These tumors are highly sensitive to radiation therapy and to combination chemotherapy, and selection of treatment therefore depends on disease stage and size of mediastinal tumor.
